# The Power of Community Engagement and Patient Voices

**DOI:** 10.1001/jamanetworkopen.2024.24790

**Published:** 2024-08-01

**Authors:** Arshiya A. Baig

**Affiliations:** Department of Medicine, University of Chicago, Chicago, Illinois.

Elsewhere in *JAMA Network Open*, Wieland et al^1^ report findings from a randomized clinical multicenter trial that assessed the use of patient digital narratives on glycemic control among Hispanic adults with type 2 diabetes. The study included a large patient population, had a high retention rate, and was conducted in primary care clinical settings in 2 states. The study team used a community-based participatory research approach to partner with community members in developing digital narratives that described individuals’ experiences living with diabetes. Patients in the intervention group watched the digital narrative video in their preferred language and were provided access to the video on their own devices. Compared with patients in the control group, those in the intervention group had a small improvement in glycemic control; the intervention was highly acceptable.

Hispanic individuals in the United States have high rates of diabetes prevalence and diabetes-related complications,^2^ with only 48% achieving adequate glycemic control.^3^ These disparities in diabetes prevalence and outcomes are multifactorial. The National Healthcare Disparities Report^4^ notes that Hispanic patients receive a lower quality of diabetes care. Structural and social determinants of health, such as access to healthy food, safe environments for exercise, and cost of medication, impede a person’s ability to manage their diabetes.^5^ Lack of access to culturally tailored and linguistically appropriate care can pose barriers to effective patient-physician communication and the receipt of self-management education and can lead to poor glycemic control.^6^ While many interventions have aimed to improve outcomes among Hispanic patients living with diabetes, many need further cultural tailoring and grounding in the community.

The use of digital narratives is a promising approach, and its engagement with community partnership in this study is novel. Community-partnered participatory research is an approach to collaborative research that emphasizes partnership with community for the design and evaluation of interventions and conduct of research studies.^7^ Recently funders, including the Patient-Centered Outcomes Research Institute and the Robert Wood Johnson Foundation, have encouraged partnership with community, stakeholders, and patients in clinical and translation studies. Engaging communities increases the likelihood that interventions are culturally tailored to patients and sustainable after the research is complete. The storytellers in this study played a key role in the production of their narratives through workshops conducted with the Rochester Healthy Community Partnership. The narratives from the workshop highlighted community members’ challenges and success in managing their diabetes. The study highlights the importance of academic and community partnership in codesigning interventions and ensuring that the stories told are ones that depict the reality of people in the community, tailored to patients’ culture, and told in their language.

Managing diabetes includes multiple daily behavioral self-management tasks, such as modifying diet and exercise habits, taking medications, checking blood glucoses, and engaging in regular health care visits. Adults with diabetes may lack support from family or friends in diabetes self-management. There is longstanding literature on the role of peer support in chronic disease management. Peers living with chronic conditions can share their knowledge, experience, and resources to support others living with diabetes. While not a direct peer support intervention, the use of digital technologies in this study provided a way for Hispanic adults living with diabetes to hear the stories of others living with the condition and how they navigated family, community, culture, and societal barriers in diabetes self-management. And as noted in the study results, the study team found that patients in the intervention group increased their confidence in managing their disease and were motivated to make behavior change after viewing the 4 narratives.

The US Hispanic population is diverse and includes people from a range of countries and with a range of educational attainment, socioeconomics, acculturation, and language preferences. Oftentimes we find that Hispanic individuals are aggregated into 1 ethnic group, which disregards this community’s rich diversity. We have seen this heterogeneity represented in diabetes prevalence: Cuban Americans have lower rates of diabetes prevalence compared with Mexican Americans and Puerto Ricans in the United States.^2^ The study team intentionally included storytellers who were women and men and those from different regions of Latin America to better reflect the richness of the US Hispanic population in their narratives. This representation of diverse voices increased the chance that the stories would resonate with individual Hispanic patients and their individual lived experience with diabetes.

The study provides evidence that a digital narrative intervention that is done in partnership with community can have an impact on clinical outcomes. The stories were developed with and for Hispanic adults with diabetes and helped normalize the challenges of living with diabetes and highlighted a way to overcome them. This digital intervention is portable and can serve as a supplement to clinical care. Future studies can further delineate the mechanisms by which digital narratives can improve diabetes self-care and impact long-term diabetes outcomes. Tailoring interventions to individual patients and their cultures is necessary to address health disparities. Patients do not live in silos. They bring with them their language, their culture, their beliefs, and their community. Addressing disparities among Hispanic patients living with diabetes involves first understanding their lives through their own authentic voices.

## References

[1] Wieland ML, Vickery KD, Hernandez V, Digital storytelling intervention for hemoglobin A_1c_ control among Hispanic adults with type 2 diabetes: a randomized clinical trial. JAMA Netw Open. 2024;7(8):e2424781. doi:10.1001/jamanetworkopen.2024.24781 39093566 PMC11297376

[2] US Department of Health & Human Services; Office of Minority Health. Diabetes and Hispanic Americans. Accessed May 14, 2024. https://minorityhealth.hhs.gov/diabetes-and-hispanic-americans

[3] Schneiderman N, Llabre M, Cowie CC, Prevalence of diabetes among Hispanics/Latinos from diverse backgrounds: the Hispanic Community Health Study/Study of Latinos (HCHS/SOL). Diabetes Care. 2014;37(8): 2233–2239. doi:10.2337/dc13-2939 25061138 PMC4113173

[4] Agency for Healthcare Research and Quality. 2023 National Healthcare Quality and Disparities Report. Accessed May 14, 2024. https://www.ahrq.gov/research/findings/nhqrdr/nhqdr23/index.html 38377267

[5] Hill-Briggs F, Adler NE, Berkowitz SA, Social determinants of health and diabetes: a scientific review. Diabetes Care. 2020;44(1):258–279. doi:10.2337/dci20-0053 33139407 PMC7783927

[6] Parker MM, Fernández A, Moffet HH, Grant RW, Torreblanca A, Karter AJ. Association of patient-physician language concordance and glycemic control for limited-english proficiency Latinos with type 2 diabetes. JAMA Intern Med. 2017;177(3):380–387. doi:10.1001/jamainternmed.2016.8648 28114680 PMC5339062

[7] Israel BA, Eng E, Schulz AJ, Parker EA. Methods in Community-Based Participatory Research for Health. Jossey-Bass; 2005:31–51.

